# Moving beyond one-size-fits-all education approaches for artificial intelligence in healthcare

**DOI:** 10.1371/journal.pdig.0001408

**Published:** 2026-05-11

**Authors:** Gemma Postill, Julie Midroni, Abhishek Moturu, Laura Rosella, Nihal Haque

**Affiliations:** 1 Temerty Faculty of Medicine, University of Toronto, Toronto, Canada; 2 Temerty Centre for Artificial Intelligence Research and Education in Medicine, University of Toronto, Toronto, Canada; 3 Institute for Health Policy Management and Evaluation, University of Toronto, Toronto, Canada; 4 Department of Computer Science, University of Toronto, Toronto, Canada; 5 Dalla Lana School of Public Health, University of Toronto, Toronto, Canada; 6 ICES, Toronto, Canada; 7 Institute for Better Health, Trillium Health Partners, Mississauga, Canada; 8 Department of Medicine, North York General Hospital, Toronto, Canada; University of Pittsburgh School of Medicine, UNITED STATES OF AMERICA

## PLOS Digital Health Opinion

Thoughtful development and use of patient-centered, high-quality artificial intelligence (AI) tools hold significant potential for improving the efficiency and quality of healthcare delivery and outcomes [1]. However, actualizing the benefits of human-AI collaboration requires improved education for healthcare providers (about AI) and for developers (about healthcare) [2,3]. Clinicians are often equipped with AI tools without sufficiently understanding them, and developers are often taught how to make models without learning how to make sense of clinical data or approach developing models for clinical applications [4–6]. Lack of awareness on how an AI model works or its limitations can lead to patient harm; just as developing a model without understanding the full clinical context can lead to patient harm [2].

Education on AI in healthcare has predominantly emphasized basic AI concepts for healthcare providers and the overall healthcare ecosystem for developers, treating all AI in healthcare as a singular entity (“one-size-fits-all” approach). Such education is usually run as short crash courses that attempt to cover the entirety of AI for healthcare providers or the whole spectrum of clinical specialties, conditions, and settings for developers [7]. These “one-size-fits-all” approaches oversimplify the subtypes of AI and their unique applications and limitations in healthcare. Improving how we conceptualize and deliver AI education to healthcare providers and to developers is paramount, as the rapid introduction of AI into clinical settings is outpacing its incorporation into medical and technical curricula.

**Fig 1:**
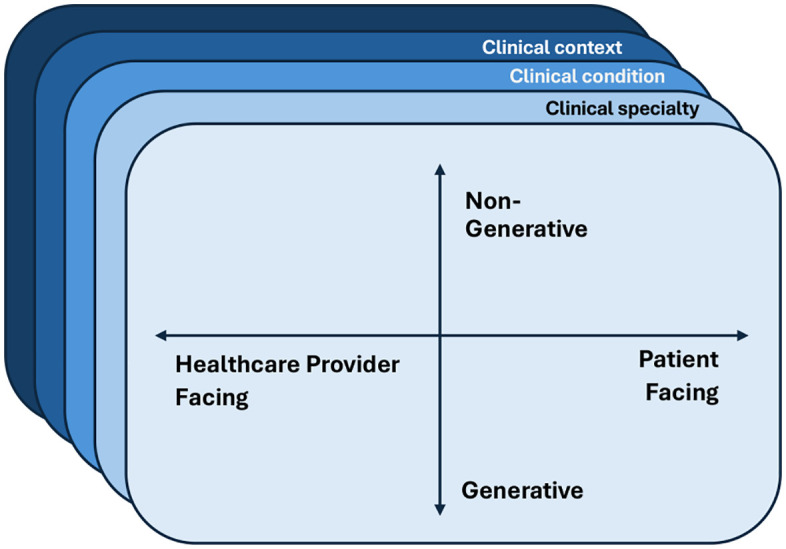
Conceptual framework illustrating the diversity of artificial intelligence (AI) tools in healthcare. To highlight variation in purpose and interaction, AI applications can be characterized along two axes: **generativity** (generative vs. non-generative) and **intended user** (healthcare provider-facing vs. patient-facing). Layers representing **clinical specialty** (e.g., emergency medicine, surgery, primary care)**, clinical condition** (e.g., appendicitis, multiple sclerosis, traumatic injury), and **clinical context** (e.g., administrative support, clinical decision making, and prognosticating outcomes) emphasize how educational needs and competencies differ across contexts. This framework underscores the importance of moving beyond one-size-fits-all approaches to AI education in healthcare.

In this opinion, we emphasize the need to move beyond one-size-fits-all discussions around AI in healthcare and towards education that recognizes the different types of AI and clinical use-cases. Further, we propose a clear, practical mental model for curriculum developers as well as their trainees (Fig 1).

## Multidimensional landscape of AI in healthcare

Educational approaches on AI in healthcare—both for healthcare providers and developers—must recognize this multidimensional landscape of healthcare AI. Applications of AI vary in their model architectures (e.g., generative or non-generative), intended users, clinical settings, clinical specialties, and clinical conditions (**Fig 1**).

In teaching AI to healthcare providers, clarifying the subtypes of AI is important. Many healthcare providers have limited formal AI education and assume all AI operates “like ChatGPT” [6,8–11]. However, AI can be generative, producing new content (e.g., language and images), or non-generative, forecasting based on learned patterns in data. The gap between what AI is and what healthcare providers perceive it to be limits both design and effective use of existing tools and highlights a focus area for AI education. It is unrealistic to suggest that healthcare providers have the same technical depth as developers. Rather, healthcare providers need to understand overarching model architecture categories and the distinct benefits, technical constraints, and liability concerns of each. For example, hallucinations (the generation of false, misleading, or nonsensical information by an AI model, often presented as if it were factual) are concerns specific to generative AI [12].

Education on AI in healthcare must also distinguish between the varying degrees of clinical oversight required by AI applications (e.g., provider-facing versus patient-facing AI tools). Provider-facing AI tools assist clinicians in making diagnoses, predictions, or treatment recommendations. Patient-facing AI systems interact directly with patients (e.g., generative AI chatbots) and have unique ethical considerations and liability concerns, particularly due to incorrect or misinterpreted outputs. As the field progresses, particularly with the rise in patient-facing AI, healthcare providers and developers must not only understand how these systems function and be able to appraise their clinical value, but also identify the legal and regulatory frameworks that govern responsibility when systems fail (e.g., European Union AI Act) [13–15].

Moving beyond the model itself, education on AI in healthcare must also cover the implications that clinical specialty, condition, and setting have on the data, as well as the downstream use, accountability, and liability associated with an AI tool (**Fig 1**). For example, ‘Na’ may reflect the elemental symbol for sodium or represent missing values in another dataset. Similarly, the application of non-generative models for administrative purposes (e.g., scheduling or resource optimization) versus prognostic models used to inform clinical decision-making entails substantially different requirements for data provenance, validation, governance, and ethical oversight. Indeed, even within a specific clinical tool, healthcare disciplines vary substantially in their goals (e.g., to stabilize, to treat, to cure, to manage patient symptoms) and workflows. Insufficient healthcare knowledge on the part of an AI developer can lead to a mismatch between a tool and its use case, or worse—inappropriate modelling assumptions that can lead to medical errors. Whilst having a large corpus of clinical knowledge is an unrealistic expectation of developers, an improved understanding of the spectrum of diversity across and within clinical specialties can equip developers with an awareness of the questions they should ask their clinical colleagues and collaborators to help ensure the relevance and application of their research. For both developers and healthcare providers, education on clinical context should be linked to the evolving AI regulations [13–15].

## Interdisciplinary collaboration for the delivery of AI in healthcare education

The concept of delivering a curriculum aligned with the proposed mental model (**Fig 1**) may appear daunting, particularly where resources or expertise may be limited. Engaging experts across diverse disciplines (e.g., computer science, medicine, epidemiology, ethics, and health policy) in the development of the curriculum avoids developing AI curricula from a single professional perspective. Centralized, interdisciplinary AI in medicine organizations (e.g., the Temerty Centre of AI Research and Education in Medicine) provide one avenue to feasibly facilitate delivery as they house and connect interdisciplinary experts, as well as share resources beyond their institution [16–18]. Small-group interdisciplinary education seminars are another option, as they can facilitate the sharing of disciplinary knowledge; developers can educate clinical colleagues on AI modalities and clinical colleagues can provide contextual understanding of health data and delineate clinical terminology (i.e., *inpatient* versus *outpatient*). Regardless of whether the intended audience is clinical, technical, or both, attention should be paid to ensuring both aspects are integrated into the curriculum so that learners gain a holistic understanding of both the technical underpinnings and real-world applications of AI. This cross-disciplinary approach not only enriches content delivery but also fosters critical thinking and contextual awareness among trainees.

## Conclusion

As the breadth of AI applications in healthcare rapidly proliferates, education on AI in healthcare, both for healthcare professionals and AI developers, must move beyond one-size-fits-all discussions around AI. Presently, this involves tailoring AI in healthcare education to the different types of AI (generative and non-generative AI), users (provider-facing and patient-facing AI), and clinical use-cases. Practically, to do so can involve collaboration with existing interdisciplinary AI in medicine organizations or leveraging multidisciplinary expertise locally. Ultimately, such education would support clinicians in critically appraising and responsibly using AI tools, while equipping developers with the clinical context and systems awareness required to design safe and meaningful applications.
